# Factors which contributed for low quality sputum smears for the detection of acid fast bacilli (AFB) at selected health centers in Ethiopia: A quality control perspective

**DOI:** 10.1371/journal.pone.0198947

**Published:** 2018-06-20

**Authors:** Ayehu Mekonen, Yeshi Ayele, Yifru Berhan, Desalegn Woldeyohannes, Woldaregay Erku, Solomon Sisay

**Affiliations:** 1 Management Science for Health, Addis Ababa, Ethiopia; 2 West HarargheZonal Health Department, TB and HIV Case Team, Chiro, Ethiopia; 3 College of Health Science, Addis Ababa University, Addis Ababa, Ethiopia; 4 Department of Animal Health and Zoonoses, Aklilu Lemma Institute of Pathobiology, Addis Ababa University, College of Health Science, Addis Ababa, Ethiopia; 5 Department of Medical Microbiology, Parasitology and Immunology, Addis Ababa University, College of Health Science, Addis Ababa, Ethiopia; 6 Federal Ministry of Health, Addis Ababa, Ethiopia; Food and Drug Administration, UNITED STATES

## Abstract

**Objectives:**

Quality of tuberculosis (TB) microscopy diagnosis is not a guarantee despite implementation of External Quality Assurance (EQA) service in all laboratories of health facilities. Hence, we aimed at evaluating the technical quality and the findings of sputum smear microscopy for acid fast bacilli (AFB) at health centers in Hararge Zone, Oromia Region, Ethiopia.

**Methods:**

A cross-sectional study was carried out between July 8, 2014 and July 7, 2015.A pre-tested structured questionnaire was used to collect data. Lot Quality Assurance Sampling (LQAS) method was put into practice for collecting all necessary sample slides. Data were analyzed by using SPSS (Statistical Package for Social Sciences) version 20 software. P-value < 0.05 was considered as statistically significant.

**Results:**

Of the total55 health center laboratories which had been assessed during the study period, 20 (36.4%) had major technical errors; 13 (23.6%) had 15 false negative results and 17 (30.9%) had 22 false positive results. Moreover, poor specimen quality, smear size, smear thickness, staining and evenness were indicated in 40 (72.7%), 39 (70.9%), 37 (67.3%), 27(49.1%) and 37 (67.3%) of the collected samples, respectively. False negative AFB findings were significantly associated with lack of Internal Quality Control (IQC) measures (AOR (Adjusted Odds Ratio): 2.90 (95% CI (Confidence Interval): 1.25,6.75) and poor staining procedures (AOR: 2.16(95% CI: 1.01, 5.11).

**Conclusions:**

The qualities of AFB smear microscopy reading and smearing were low in most of the laboratories of the health centers. Therefore, it is essential to strength EQA program through building the capacity of laboratory professionals.

## Introduction

Tuberculosis(TB) is a major public health problem throughout the world [1], rated to be the second leading cause of death from an infectious disease worldwide [2]. About one-third of the world’s population is estimated to be infected with tubercle bacilli, and hence at risk of developing active disease. According to the 2017global TB report of world health organization (WHO), 6.3 million people were estimated to be infected with TB in 2016alone, while an estimated 1.3 million people died due to TB infection among non-HIV infected individuals [1]. Additionally, an estimated 4.1% of new TB cases and 19% of previously treated cases were believed to have multi drug resistant (MDR)-TB whereas, an estimated 240, 000 people died of MDR-TB in the same year [1–2].

TB still remains to be the second leading cause of death from an infectious disease worldwide [2].Ethiopia is among the 22 high TB and the 27 high MDR-TB burden countries [1]. According to 2010/11 national TB prevalence survey, the prevalence of smear-positive TB among adults was 108 cases per 100,000 populations [2].

Direct light microscopy for acid-fast bacilli (AFB) is one of the most widely used and accepted diagnostic and monitoring tools for TB infection at peripheral health facilities [3]. In the End TB strategy of WHO, quality-assured TB microscopy service is still one of the key elements of Directly Observed Treatment Short-course (DOTS)[1–3]. However, the quality of TB diagnostic service has a major influence on the monitoring of the progression of TB control program as false microscopy results could lead to failure in detecting TB patients, unnecessary treatment of non-TB cases and development of MDR-TB [4].

Quality assurance consists of quality control (QC), external quality assurance (EQA), and quality improvement (QI)components, which are essential tools to yield reliable and reproducible laboratory results [5]. Reliable AFB microscopic results (such as smear positivity rates) also help program officers to assess the progress of TB control activities [6–7].

Reliable laboratory service provides results that are consistently accurate [8]. At the same time, the reliability of the diagnosis can be ensured through commitment to quality assurance service. Therefore, it is essential to periodically implement EQA as a key component of quality assurance program for ensuring the quality of sputum smear microscopy in DOTS implementing health facilities [5]. In Ethiopia, implementation of quality assurance schemes in regards to sputum smear microscopy has been in practice at all public health laboratories to improve and sustain the quality of monitoring of the national TB control program[6].

However, the existence of EQA alone is not a warrant for enhancing case detection of all forms of TB to meet WHO’s target of 70% detection rate [5]. In fact, the case detection of all forms of TB was documented to below (50%) in West Hararghe Zone of Oromia Region, despite the implementation of EQA service in those DOTS providing health facilities [9].This signified the need to check the quality of sputum smear microscopy. Therefore, the aim of this study was to evaluate the quality of sputum smear microscopy among selected public health center laboratories in the study area.

## Methods

### Study design

A cross-sectional study was conducted from July 8, 2014 up to July 7,2015 at selected public health center laboratories in West Hararghe Zone, Oromia Region, Ethiopia.

### Study setting

According to the 2007 census projection, the total population of the Zone was estimated to be 1,871,706. Of which, 958,861 (51.2%) were men and the rest women [10].A total of 75 public health facilities were found in West Hararghe Zone [9] and out of which, only 55 health facility laboratories (which were included in this study) provided AFB diagnostic services. Ziehl Neelsen (ZN) and florescent microscopy (FM) methods were used to diagnose TB infection. Two public hospitals and 65 public health centers were providing sputum smear microscopy diagnostic services in the study area [11]. EQA program was decentralized to 2 public hospital EQA centers since 2012 [11].Quarterly blind rechecking and semiannual onsite evaluation were conducted by the centers for implementing EQA activities to their catchment area of health facilities.

### Study population

All health facilities which were found in the West Hararghe Zone were considered as the source population.

### Study subject

All health centers which were providing AFB diagnostic services were considered as study subject.

### Sample size and sampling method

All laboratories which kept all AFB slides for blind rechecking under EQA program, and those laboratories which reported positive slides based on WHO grading system were enrolled in this study. LQAS method was put into practice for collecting the required number of AFB slide samples with assumption of 95% CI, zero acceptance number, 100% of specificity and 80%of sensitivity as per the national EQA guideline [12].

### Exclusion criteria

Those laboratories which were utilizing FM for pulmonary TB diagnosis were excluded from the study.

### Data collection procedure and quality assurance

A pre-tested questionnaire was utilized by trained district TB officers and laboratory professionals for collecting the necessary information. The collected slides were transported to two EQA centers. At each EQA center, slides were blindly rechecked by trained initial controller. First controller coded and read using 100 × objectives. Discordant slides were re-read by second reader (controller). Discrepant readings between the peripheral laboratory reading and the second reader were re-read and verified by third controller.

### Operational definitions

Performance of sputum smear microscopy was considered as good when the laboratories had no major errors [4].Specimen qualities, smear staining, size, thickness, evenness and cleaning were used as smear quality indicators. Laboratories which scored 80% and above in smear quality indicators were taken as good smear performers [6].The operational definition of reading error types was based on the national EQA guideline [4–6].

1. High False Negative (HFN):misread 1+ to 3+ positive smears as negative.
2. High False Positive (HFP):misread1+ to 3+negative smears as positive.
3. Low False Positive (LFP): misread a scanty negative smear as (1 - 9AFBs/100 fields) as positive.
4. Low False Negative (LFN): misread a scanty positive smear (1 - 9AFBs/100 fields) as negative.
5. Quantification Error (QE):difference of more than one grade in reading a positive slide between examinee and controller.
6. Major Error: smear result with HFN or HFP.
7. Minor Error: smear result with LFN or LFP or QE.

### Data analysis

Data were entered into EPI info version 7software and exported into SPSS version 20 software for analysis. Smear quality indicators and AFB results were calculated. Each laboratory was evaluated for major errors, minor errors, and false negative and false positive results. Variables, which had P-value ≤ 0.2 in bivariate analysis, were entered into multivariate analysis to control confounding factors. P-value < 0.05 was considered as statistically significant.

### Ethics approval

The study was conducted after obtaining ethical approval from Ethiopian Public Health Institute (EPHI). Sputum smear slides which were collected through routine clinical practice were randomized for re-blind checking (RBC). Data for the study were obtained through routine laboratory quality monitoring system, but were not directly collected from patients. The sputum smear slides had no patient identification information and the results were reported to evaluate the laboratory performance, but not to directly link patient for treatment. West Hararghe Zonal Health Department provided the permission to conduct the study. Results were kept confidential and communicated to only respective laboratories.

## Results

### General characteristics

A total of 55 laboratories were enrolled in this study. Of these, 54 (98.2%) of the laboratories had trained laboratory professionals. Besides, 24 (43.6%) and 21 (38.2%) of the laboratories were supplied with main electric power and running tap water, respectively. Twelve (21.8%) of the laboratories had separate room for TB microscopy. Similarly, 12 (21.8%) of laboratories used the recommended lens tissue and solutions to clean their microscopes. All of the studied laboratories were regularly supervised in EQA program. Out of these, 17 (30.9%) did not receive EQA feedback reports (Table 1).

**Table 1:** General characteristics of the studied laboratories in West Hararghe Zone, Oromia Region, 2015.

Variables	Out comes	Frequency	Percentage
Separate microscope for TB	Yes	12	21.8
	No	43	78.2
Running water in the laboratory	Yes	21	38.2
	No	34	61.8
Microscope light source	Mirror	3	5.5
	Electric power	24	43.6
	Solar	28	50.9
Preventive maintenance for microscopy	Yes	20	36.4
	No	35	63.6
Clean microscope by	DEE with lens tissue	12	21.8
	70% of alcohol with lens tissue	30	54.5
	70% of alcohol with cotton/gauze	13	23.6
Trained laboratory professionals	Yes	54	98.2
	No	1	1.8
Participated in EQA program	Yes	55	100
	No	0	0
Received feedback	Yes	38	69.1
	No	17	30.9
IQC measure	Yes	12	16.4
	No	43	83.6
Job aids available	Yes	19	34.5
	No	36	65.5
Irregular supply of sputum cup	Yes	41	74.5
	No	14	25.5
Irregular supply of reagent	Yes	46	83.6
	No	9	16.7

TB: Tuberculosis; DEE: Di Ethyl Ether; EQA: External Quality Assessment; IQC: Internal Quality Control

### Reagents, supplies and internal quality control (IQC) measures

Forty-six (83.6%) of the 55 EQA program participant laboratories were not regularly supplied with AFB reagents. Cleaning microscope by diethyl ether with lens tissue, 70% of alcohol with lens tissue and70% of alcohol with cotton/gauze were being practiced in 12 (21.8%), 30 (54.5%) and 13 (23.6%) of the laboratories, respectively. Only 9(16.9%) of the laboratories did weekly internal quality control measures for new AFB reagents by comparing known positive and negative control smears (Table 1).

### Annual negative slide volume and slide positivity rate

Twenty-six (47.3%) of the participating laboratories had low slide positivity rate (SPR) for AFB slides. Moreover, 12 (21.8%) of laboratories had low annual negative slide volume (ANSV) (< 301 slides per year).Furthermore, both low SPR and low ANSV were found in 7(12.7%) of the laboratories (Table 2).

**Table 2:** ANSV and SPR of smear microscopy among the studies laboratories in West Hararghe Zone, Oromia Region, 2015.

Number of slides	SPR < 5%	SPR = 5% - 10%	SPR > 10%	Total
ANSV	N (%)	N (%)	N (%)	N (%)
<301 slides	7 (12.7)	5 (9.1)	0 (0.0)	12 (21.8)
301–500 slides	7 (12.7)	7 (12.7)	2 (3.6)	16 (37.3)
501–1000 slides	12 (21.8)	13 (23.6)	2 (3.6)	27 (13.9)
>1000 slides	0 (0.0)	0 (0.0)	0 (0.0)	0 (1.5)
Total	26 (47.3)	25 (45.6)	4 (7.2)	55 (100.0)

ANSV: Annual Negative Slide Volume; SPR: Slide Positivity Rate; N: Number

### Specimen, staining and smear quality of AFB slides

**Fig 1:**
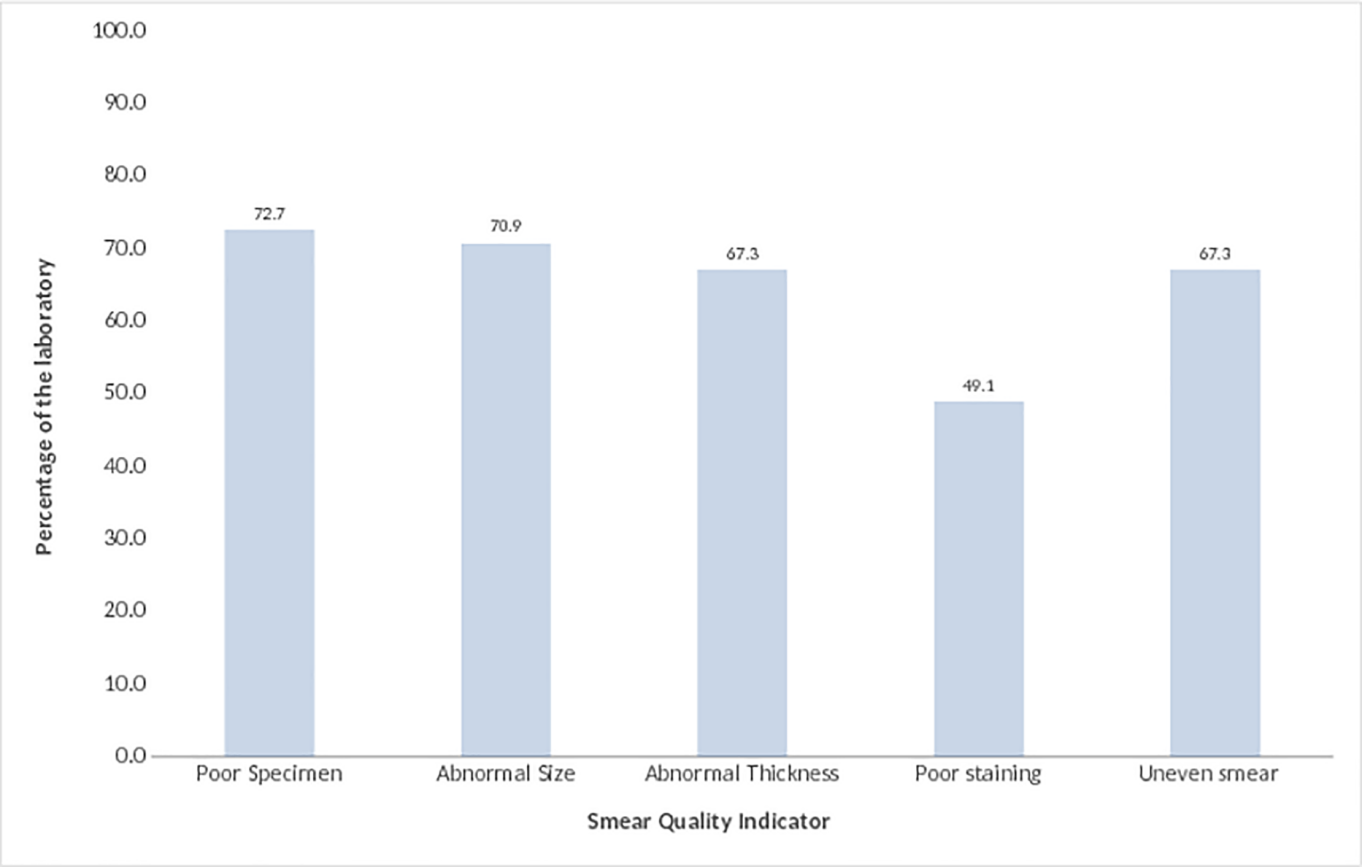
Performance of specimen quality, staining and smearing techniques among laboratories in West Hararghe Zone, 2015.

Of the4981blindly rechecked slides, poor specimen qualities were found in 3621(72.7%) slides, namely, 2445(49.1%) of slides were poorly stained, and 3531 (70.9%) of them had abnormal smear size. Both abnormal thickness and uneven of smear of specimen were observed in 3352(67.3%)of the slides. Overall, none of the 55 laboratories had fulfilled the overall six indicators of smearing quality (Fig 1).

In the study, those participating laboratories, which had essential supplies and engaged in EQA program, had low proportion values (Table 3) for measure of indicators for poor quality of smear (or sputum quality) and poor microscopy performance as compared to those laboratories who had not.

**Table 3:** Comparison of the characteristics of the studied laboratories with poor quality of sputum sample and poor microscopy service in West Hararghe Zone, Oromia Region, 2015.

Variables	Out comes	Poor sputum quality (smear) (N(%))					Poor microscopy performance (N(%))							
		Specimen	Size	Thickness	Staining	Eveness	LFN	HFN	QE	LFP	HFP	Minor Error	Major Error	
Separate microscope for TB (N = 12)	Yes	2(16.7)	4(33.4)	2(16.7)	------	------	6(50)	------	------	5(41.7)	------	6(50)	------	
	No	10(83.3)	8(66.6)	10(83.3)	12(100)	12(100)	6(50)	12(100)	12(100)	7(58.3)	12(100)	6(50)	12(100)	
Running water in the laboratory (N = 21)	Yes	6(28.6)	7(33.3)	8(38.1)	------	7(33.3)	6(28.6)	------	------	7(33.3)	------	6(28.6)	------	
	No	15(71.4)	14(66.7)	13(61.9)	21(100)	14(66.7)	15(71.4)	21(100)	21(100)	14(66.7)	21(100)	15(71.4)	21(100)	
Preventive maintenance (N = 20)	Yes	5(25)	7(35)	7(35)	------	7(35)	6(30)	------	------	7(35)	------	6(30)	------	
	No	15(75)	13(65)	13(65)	20(100)	13(65)	14(70)	20(100)	20(100)	13(65)	20(100)	14(70)	20(100)	
Training (N = 54)	Yes	15(27.8)	16(29.6)	17(31.5)	26(48.1)	19(35.2)	6(11.1)	7(12.9)	3(5.6)	7(13)	6(11.1)	6(11.1)	19(35.2)	
	No	39(72.2)	38(70.4)	37(68.5)	28(51.9)	35(64.8)	48(88.9)	47(87.1)	51(94.4)	47(87)	48(88.9)	48(88.9)	35(64.8)	
Participated in EQA program (N = 55)	Yes	15(27.2)	16(29.1)	17(30.9)	27(49.10	16(29.1)	6(10.9)	7(12.7)	4(7.3)	7(12.7)	7(12.7)	6(10.9)	20(36.4)	
	No	40(72.8)	39(70.1)	38(69.1)	28(50.9)	39(70.1)	49(89.1)	48(87.3)	51(92.7)	48(87.3)	48(87.3)	49(89.1)	35(63.6)	
Received feedback (N = 38)	Yes	15(39.5)	13(34.2)	16(42.1)	10(26.3)	18(47.4)	6(15.8)	------	------	)7(18.4)	------	6(15.8)	3(7.9)	
	No	23(60.5)	25(65.8)	22(57.9)	28(73.7)	20(52.6)	32(84.2)	38(100)	38(100)	31(81.6	38(100)	32(84.2)	35(92.1)	
IQC measure (N = 12)	Yes	1(8.3%)	4(33.3)	2(16.7)	----	----	5(41.7)	----	----	3(25)	----	5(41.7)	----	
	No	11(91.7)	8(66.7)	10(83.3)	12(100)	12(100)	7(58.3)	12(100)	12(100)	9(75)	12(100)	7(58.3)	12(100)	
Job aids available (N = 19)	Yes	4(21.1)	7(36.8)	6(31.6)	-----	6(31.6)	6(31.6)	-----	-----	7(36.8)	-----	6(31.6)	-----	
	No	15(78.9)	12(63.2)	13(68.4)	19(100)	13(68.4)	13(68.4)	19(100)	19(100)	12(63.2)	19(100)	13(68.4)	19(100)	
Irregular sputum cup supply (N = 41)	Yes	40(97.6)	29(70.7)	35(85.4)	27(65.9)	34(82.9)	------	34(82.9)	37(90.2)	40(97.6)	34(82.9)	------	20(48.8)	
	No	1(2.4)	12(29.3)	6(14.6)	14(34.1)	7(17.1)	41(100)	7(17.1)	4(9.8)	1(2.4)	7(17.1)	41(100)	21(51.2)	
Irregular reagent supply (N = 46)	Yes	39(84.8)	34(73.9)	34(73.9)	27(58.7)	33(71.7)	44(95.6)	39(84.8)	42(91.3)	42(91.3)	39(84.8)	44(95.6)	20(43.5)
	No	7(15.2)	12(26.1)	12(26.1)	19(41.3)	13(28.3)	2(4.4)	7(15.2)	4(8.7)	4(8.7)	7(15.2)	2(4.4)	26(56.5)

TB: Tuberculosis; EQA: External Quality Assessment; IQC: Internal Quality Control; LFN: Low False Negative; HFN: High False Negative; QE: Quantification Error; LFP: Low False Positive; HFP: High False Positive; N: Number

### Sputum smear microscopic performance

**Fig 2:**
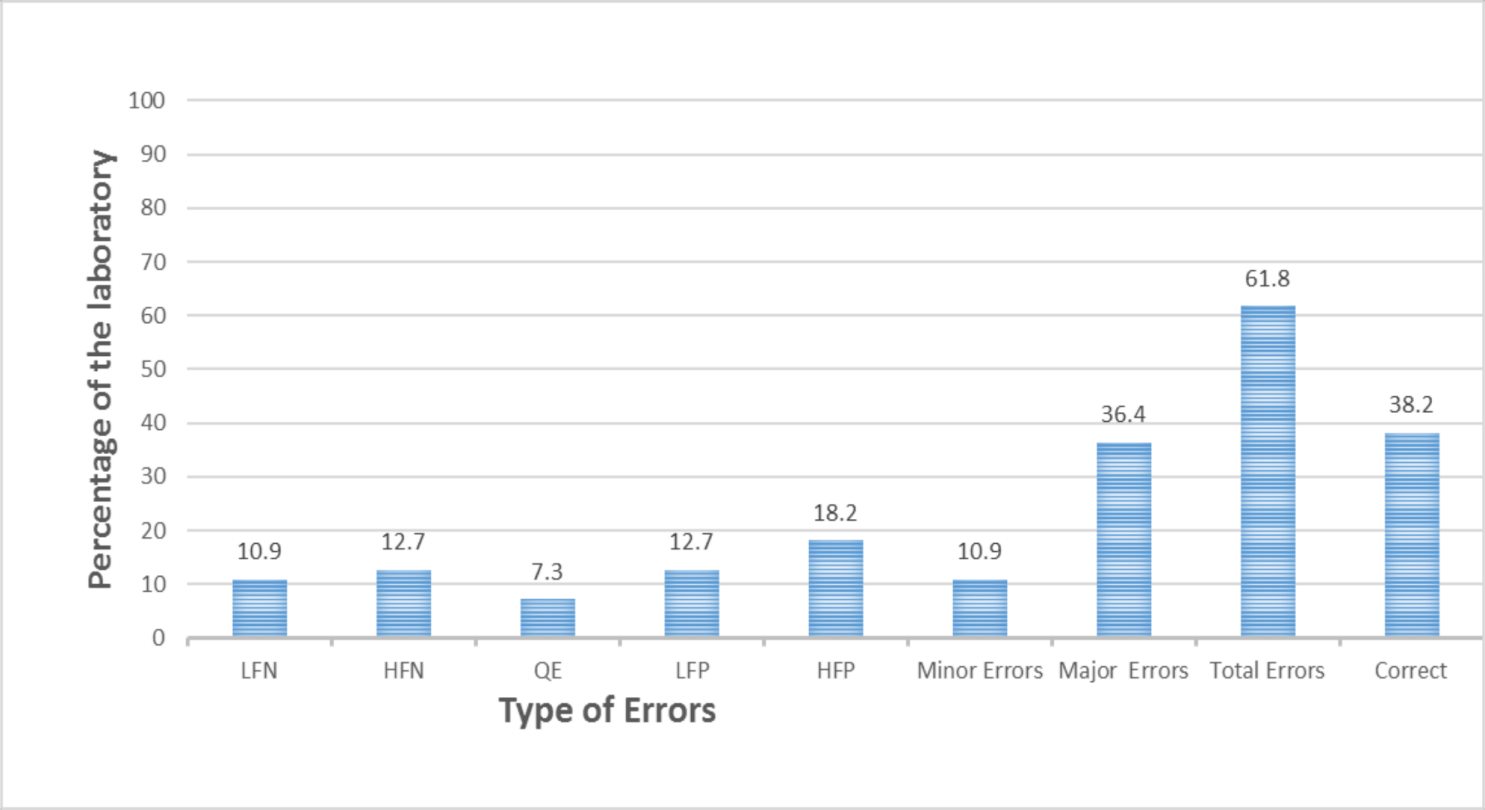
Performance of sputum smears microscopy among the studied laboratories in West Hararghe Zone, Oromia Region, 2015. LFN: Low False Negative; HFN: High False Negative; QE: Quantification Error; LFP: Low False Positive; HFP: High False Positive.

Majority of the laboratories (34(61.8%)) had at least one microscopy error (a total of 22 false positive, 15 false negative and 4 quantification errors). At least one major error was found in 20(36.4%) of the laboratories. Of the 34 laboratories which had discordant results, 17(40%) and 13 (23.6%) had false positive and false negative results, respectively (Fig 2).

### Factors for false negative and false positive results

Absence of IQC measures, poor microscope smear, poor smear thickness, poor evenness and size of smear were significantly associated with false negative results. Moreover, laboratories with poor staining procedures had more likely to have false negative results (9(33.3%) (AOR: 2.16; 95% CI: 1.01, 5.11)) as compared to those with good staining procedures(4 (14.3%)) (Table 4). At the same time, poor smear thickness resulted in having high chance of false negative results (AOR: 2.65; 95% CI: 1.14, 6.18) as compared to those laboratories with good smear thickness. Those laboratories with no IQC measures had significantly higher false negative results (AOR: 2.90; 95% CI: 1.25, 6.75).

**Table 4:** Factors for false negative smear microscopy results among the studied laboratories in West Hararghe Zone, Oromia Region, 2015.

Variables		False negative		COR (95%CI)	AOR (95%CI)	P- value
		No	Yes			
Separate microscope	No	8	4	1.01(0.83, 1.23)	------	
	Yes	34	9	1	------	
IQC practice	No	32	11	2.43 (1.09, 5.44)	2.90 (1.25, 6.75) **	0.013
	Yes	10	2	1	1	
Preventive maintenance	No	27	8	1.06(0.85, 1.21)	1.02(0.85, 1.26)*	0.021
	Yes	15	5	1	1	
Good specimen quality	No	30	10	0.87(0.78, 0.98)	------	
	Yes	12	3	1	------	
Good smear size	No	30	9	1.05(0.86, 1.30)	1.05(0.87, 1.33)*	0.005
	Yes	12	4	1	1	
Good smear thickness	No	28	10	1.06 (0.86, 1.29)	1.04 (0.69, 1.58)*	0.023
	Yes	14	3	1	1	
Good staining	No	18	9	2.50 (1.12, 5.58)	2.16 (1.01, 5.11) **	0.012
	Yes	24	4	1	1	
Evenness of the smear	No	27	10	1.04(1.03, 1.05)	1.22(0.31, 4.88)*	0.034
	Yes	15	3	1	1	

IQC: Internal Quality Control; COR: Crude Odds Ratio; AOR: Adjusted Odds Ratio; CI: Confidence Interval

NB** = significant (P<0.05).

Adjusted*: for separate microscope, IQC practice, preventive maintenance, specimen qualities, smear size, smear thickness, staining and evenness of the smear.

On the other hand, absence of IQC measures had also statistically significant association with false positive results: those laboratories which lacked IQC measures were more likely to have false positive results (AOR: 3.43; 95% CI: 1.39, 8.45) as compared to those having IQC measures (Table 5).

**Table 5:** Factors for false positive smear microscopy results among the studied laboratories in West Hararghe Zone, Oromia Region, 2015.

Variables		False positive		COR (95%CI)	AOR (95%CI)	P- value
		No	Yes			
Separate microscope	No	30	13	1.83(0.83, 4.07)	2.20 (0.94, 5.16)*	0.071
	Yes	8	4	1	1	
IQC practice	No	29	14	2.57(1.11, 5.97)	3.43 (1.39, 8.45) **	0.007
	Yes	9	3	1	1	
Preventive maintenance	No	25	10	2.54(0.90, 7.16)	2.74 (0.93, 8.06)*	0.027
	Yes	13	7	1	1	
Good specimen quality	No	31	9	0.2 (0.1, 0.4)	0.3 (0.2, 0.5) **	0.009
	Yes	7	8	1	1	
Good smear size	No	29	8	2.57 (0.99, 6.63)	------	
	Yes	9	7	1	------	
Good smear thickness	No	25	13	2.94 (1.35, 6.40)	2.65 (1.14, 6.18) **	0.014
	Yes	13	4	1	1	
Good staining	No	18	9	0.02 (0.01, 0.07)	0.02 (0.01, 0.1) **	0.002
	Yes	20	8	1	1	
Evenness of the smear	No	26	11	1.16(0.84, 1.60)	1.16(0.84, 1.56)*	0.047
	Yes	12	6	1	1	

IQC: Internal Quality Control; COR: Crude Odds Ratio; AOR: Adjusted Odds Ratio; CI: Confidence Interval

NB** = significant (P<0.05).

Adjusted*: for separate microscope, IQC practice, preventive maintenance, specimen quality, smear size, smears thickness, staining and evenness of the smear.

## Discussion

Sputum smear examination for the detection of AFB in the diagnosis of TB stays a key technique in resource poor settings. However, poor quality of diagnosis results in a lower TB case detection, and hence predisposes the community to active transmission. Likewise, poor laboratory quality performance also results in false positive TB diagnosis which leads to unnecessary treatments. Overall, we found that the existence of poor sputum smear quality in those health facilities which had been assessed for evenness, thickness, smearing and staining procedures.

Out of the total 55 laboratories included in the study, 20(36.4%)had major errors such as high false positive and high false negative results. This might indicate the technical gaps among the laboratory professionals in the study area. This finding is relatively lower as compared to a result found in Democratic Republic of Congo (61.5%) [7]; however, it was relatively higher than reports from another study in Eastern part of Ethiopia (3.8%)[13] and New Delhi in India (16.7%)[14]: these discrepancies might be due to the methodological differences such as blind rechecking method versus panel testing method which was implemented by the other studies. Moreover, it could indicate the influence of the country’s policy on laboratory quality assurance procedure to be followed.

In this study, false positive results were found in 22 (40%) of the laboratories. These results could lead patients to start unnecessary treatment, wastage of valuable medication, cause emotional trauma to patients and their families [2–3] [5]. The high false positive rate found in this study is higher compared to another studies conducted in Ethiopia [15], Tanzania [16], India [17] and Iran [18].The high false positive results in this study were significantly higher among laboratories which lacked IQC measures and poor staining procedures. This implicates that IQC and appropriate staining practices are essential tools for reducing false positive results. At the same time, strict follow up of EQA program was paramount important to maintain the quality of AFB diagnostic service, to identify root causes of problems, to provide possible solutions and avoid the recurrence of problems [7].

This study also showed that those participating laboratories, which had necessary supplies and incorporated in EQA program, had better performance (Table 3) in terms of maintaining quality of sputum smearing and microscopy service for providing reliable AFB diagnostic service to the community. It is, therefore, essential to monitor and follow all TB diagnosis laboratories as per the standard of the Ethiopian Public Health Institute AFB and EQA manual to ensure the smooth implementation of the service through capacity building of laboratory professionals; provision of essential laboratory supplies and other basic infrastructural services [6].

Low SPR and ANSV are indicators of poor sputum smear microscopic performance [6]. In this study, 26(47.3%) of laboratories were found to have below 5% of SPR. Both low SPR and ANSV were found in 7 (12.7%) of the participant laboratories. These findings were relatively lower as compared to the report from a study done in West Amhara Region of Ethiopia [15] which showed that 47% of diagnostic centers were found to have low SPR (< 5%) and ANSV (< 301 slides). On the contrary, rate of low SPR and ANSV from this study was higher than that reported from a study done in New Delhi [14] which revealed that 2.9% of participating diagnostic centers had both low SPR (< 5%) and ANSV (< 301 slides). WHO’s recommendation for laboratories with such poor performance is to intensively assess their performances in order to minimize false negative or false positive results or should discontinue providing AFB diagnostic service if they wouldn’t be improved otherwise [5].

In the study, the false negative results in 13 laboratories results could attribute to the spread of TB to family and community, and even death, which was also the case in another study [5]. This finding was higher as compared to other similar studies done in India [18], Iran [7] and Taiwan [19].

This study revealed poor specimen quality, poor smear size, poor smear thickness, poor staining and evenness, which had all higher rate than those reported from the studies done in West Amhara Region in Ethiopia [15] and Taiwan [19].This could mean that the performance of the laboratories was poorer as compared to 80% of smear quality cut-off value set by the Ethiopian national guideline [6]. Unlike to this, an acceptable specimen quality and preparation of smears were reported in studies carried out in Argentina [20] and Tanzania [16].This is regardless of the fact that such poor smears could cause false negative results [18].The absence of salivary specimen rejection in human immune deficiency virus (HIV) infected patients may also affect the smears evenness, thickness and specimen quality [7].

### Limitation of the study

The study did not include proficiency testing due to budget constraint.

## Conclusions

The overall performance of the laboratories included in the study for smear microscopy reading and smear quality was poor. Moreover, absence of IQC measures and poor smearing techniques were some of the risk factors for findings of false positive or negative results. Therefore, it is essential to strength both EQA and IQC programs and build the capacity of laboratory professionals on smear quality indicators for ensuring quality diagnosis of AFB through appropriate sputum smearing technique.

## Abbreviations

AFB: Acid Fast Bacilli
AOR: Adjusted Odds Ratio
ANSV: Annual Negative Slide Volume
CI: Confidence Interval
DOTS: Directly Observed Treatment Short-course
DEE: Di Ethyl Ether
FM: Fluorescent Microscope
EPHI: Ethiopian Public Health Institute
EQA: External Quality Assessment
HFN: High False Negative
HFP: High False Positive
LFP: Low False Positive
HIV: Human Immune Deficiency Virus
LFN: Low False Negative
IQC: Internal Quality Control
LQAS: Lot Quality Assurance Sampling
MDR-TB: Multi Drug Resistant Tuberculosis
QE: Quantification Error
QI: Quality Improvement
RBC: Re-Blind Checking
SPR: Slide Positivity Rate
SPSS: Statistical Package for Social Sciences
WHO: World Health Organization
ZN: Ziehl Neelsen

## References

[1] World Health Organization. Global tuberculosis report. Geneva, Switzerland; 2017.

[2] Federal Ministry of Health. Guidelines for clinical and programmatic management of TB, TB/HIV and leprosy in Ethiopia, 6thedition. Addis Ababa, Ethiopia; 2016.

[3] Melese M, Jerene D, Alem G, Seid J, Belachew F, Kassie Y, Habte D, Negash S, Ayana G, Girma B, Haile YK, et al Decentralization of acid fast bacilli (AFB) external quality assurance using blind rechecking for sputum smear microscopy in Ethiopia. PloS ONE. 2016; 18;11(3):e0151366 doi: 10.1371/journal.pone.0151366 26991651 10.1371/journal.pone.0151366 PMC4798724

[4] World Health Organization. Guidelines for treatment of tuberculosis. 4thedn. M/TB/2009.420. Geneva, Switzerland; 2010.

[5] World Health Organization. Global tuberculosis report. Geneva, Switzerland; 2012.

[6] Ethiopian Health and Nutrition Research Institute. Guidelines for quality assurance of smear microscopy for tuberculosis diagnosis. Addis Ababa, Ethiopia;2009.

[7] Rie A, Fitzgerald D, Kabuya G, Deun A, Tabala M, Jarret N, et al Sputum smears microscopy: Evaluation of impact of training, microscope distribution, and use of external quality assessment guidelines for resource-poor settings. J Clin Micro Biol. 2008;46(3): 897–901. 10.1128/JCM.01553-07 PMC2268372 18174302

[8] Simsek H. Requirement of quality assessment for modern tuberculosis laboratory services. Mycobact Diseases. 2011; 1(1):2.

[9] Oromia Regional Health Bureau. Tuberculosis annual report of West Hararghe Zonal Health Department. Chiro, Ethiopia; 2014.

[10] Central Statistical Agency. The 2007 population and housing census of Ethiopia; 2008.

[11] Oromia Regional Health Bureau. Tuberculosis annual report. Addis Ababa, Ethiopia; 2013.

[12] Ethiopian Health and Nutrition ResearchInstitute.AFB smears microscopy manual. Addis Ababa, Ethiopia; 2009.

[13] Ayana DA, Kidanemariam ZT, Tesfaye HM, Milashu FM.External quality assessment for acid fast bacilli smears microscopy in Eastern part of Ethiopia. BMC Res Notes. 2015; 8:537 doi: 10.1186/s13104-015-1478-0 26437958 10.1186/s13104-015-1478-0 PMC4593188

[14] Jha K, Thapa B, Salhotra V, Afridi N. Panel testing of sputum smear microscopy of national tuberculosis reference laboratories in SAARC Region. SAARC J Tuber Lung Dis.2011; 8(1): 20–30.

[15] Shiferaw MB, Hailu HA, Fola AA, Derebe MM, Kebede AT, Kebede AA, Emiru MA, Gelaw ZD, et al Tuberculosis laboratory diagnosis quality assurance among public health facilities in West Amhara Region, Ethiopia. PloS ONE. 2015; 16; 10(9):e013848. 10.1371/journal.pone.0138488 PMC4574440 26376438

[16] Basra D, Matee M, Mcnerney R. Quality assessment of sputum smears microscopy for detection of Acid fast bacilli in peripheral health care facilities in Dare-selam, Tanzania. East Afr Med J.2006; 83(6): 306–310. 16989375 10.4314/eamj.v83i6.9437

[17] Peter TF, Rotz PD, Blair DH, Khine AA, Freeman RR, Murtagh MM, et al Impact of laboratory accreditation on patient care and the health system. Am J Clin Pathol. 2010; 134:550–555. doi: 10.1309/AJCPH1SKQ1HNWGHF 20855635 10.1309/AJCPH1SKQ1HNWGHF

[18] Selvakumar N, Murthy B, Prabhakaran E, Sivagamasundari S, Vasanthan S, Perumal M, et al Lot Quality Assurance (LQA) sampling of sputum acid-fast bacillus smears for assessing sputum smear microscopy centers. J Clin Micro Biol. 2005; 43(2): 913–915. 10.1128/JCM.43.2.913-915.2005 PMC548059 15695704

[19] Wu M, Chiang C, Jou R, Chang S, Luh K. External quality assessment of sputum smears microscopy in Taiwan. Int J Tuber Lung Dis. 2009; 13(5): 606–612. 19383194

[20] Ozkutuk A. Quality assurance in tuberculosis laboratories. Micro Biol Bul.2009; 43: 699–707. 20084926

